# Identification of a Four-Gene-Based SERM Signature for Prognostic and Drug Sensitivity Prediction in Gastric Cancer

**DOI:** 10.3389/fonc.2021.799223

**Published:** 2022-01-12

**Authors:** Xiya Jia, Bing Chen, Ziteng Li, Shenglin Huang, Siyuan Chen, Runye Zhou, Wanjing Feng, Hui Zhu, Xiaodong Zhu

**Affiliations:** ^1^Department of Medical Oncology, Fudan University Shanghai Cancer Center, Shanghai, China; ^2^Department of Oncology, Shanghai Medical College of Fudan University, Shanghai, China

**Keywords:** gastric cancer, cancer stem cells, epithelial-mesenchymal transition, prognostic signature, drug sensitivity prediction

## Abstract

**Background:**

Gastric cancer (GC) is a highly molecular heterogeneous tumor with poor prognosis. Epithelial-mesenchymal transition (EMT) process and cancer stem cells (CSCs) are reported to share common signaling pathways and cause poor prognosis in GC. Considering about the close relationship between these two processes, we aimed to establish a gene signature based on both processes to achieve better prognostic prediction in GC.

**Methods:**

The gene signature was constructed by univariate Cox and the least absolute shrinkage and selection operator (LASSO) Cox regression analyses by using The Cancer Genome Atlas (TCGA) GC cohort. We performed enrichment analyses to explore the potential mechanisms of the gene signature. Kaplan-Meier analysis and time-dependent receiver operating characteristic (ROC) curves were implemented to assess its prognostic value in TCGA cohort. The prognostic value of gene signature on overall survival (OS), disease-free survival (DFS), and drug sensitivity was validated in different cohorts. Quantitative reverse transcription polymerase chain reaction (RT-qPCR) validation of the prognostic value of gene signature for OS and DFS prediction was performed in the Fudan cohort.

**Results:**

A prognostic signature including SERPINE1, EDIL3, RGS4, and MATN3 (SERM signature) was constructed to predict OS, DFS, and drug sensitivity in GC. Enrichment analyses illustrated that the gene signature has tight connection with the CSC and EMT processes in GC. Patients were divided into two groups based on the risk score obtained from the formula. The Kaplan-Meier analyses indicated high-risk group yielded significantly poor prognosis compared with low-risk group. Pearson’s correlation analysis indicated that the risk score was positively correlated with carboplatin and 5-fluorouracil IC50 of GC cell lines. Multivariate Cox regression analyses showed that the gene signature was an independent prognostic factor for predicting GC patients’ OS, DFS, and susceptibility to adjuvant chemotherapy.

**Conclusions:**

Our SERM prognostic signature is of great value for OS, DFS, and drug sensitivity prediction in GC, which may give guidance to the development of targeted therapy for CSC- and EMT-related gene in the future.

## Introduction

Gastric cancer (GC) is one of the most common malignant tumors which have high morbidity and mortality, and it is the fourth leading cause of cancer-related death. A total of 1,089,103 people were diagnosed with GC worldwide in 2020, and new deaths increased to 768,793 which accounted for 7.7% of cancer-related death (1, 2). Although overall GC incidence rates continue to decrease in the majority of countries, including high-incidence countries such as China, Korea, and Japan, the absolute number of newly diagnosed GC cases and the incidence in younger age groups (below age 50 years) are predicted to continue to increase in both low- and high-risk countries (3). Nowadays, different classification and staging systems such as TNM staging, Lauren classification, and Borrmann classification are extensively used to predict the outcomes and plan personalized treatment strategies for GC patients in clinical practice. However, the outcomes can vary significantly for the patients with similar clinicopathological characteristics because molecular heterogeneity has been shown in similar stages and classifications, suggesting the current classification system is insufficient to achieve precise prognostication and risk stratification. Hence, novel strategies providing more precise predictive value are strongly demanded for making individualized treatment strategies.

Recently, some literature reports that the molecular heterogeneity (gene expression, gene amplification, epigenetic changes, chromosomal aberrations) between GC patients can be used to develop molecular classification systems to stratify patients to different molecular subtypes with different outcomes. The Asian Cancer Research Group (ACRG) proposed a molecular classification containing 4 molecular subtypes: MSS/TP53 activation, MSS/TP53 loss, microsatellite instability (MSI), and MSS/EMT. The result of survival analysis between different molecular subtypes illustrated that the MSI group had a better prognosis and the MSS/EMT group had the worst prognosis (4). Next-generation sequencing for tumor tissue has been widely used in clinical practice to detect genetic alterations. The most widely used molecular classification of GC is based on the human epidermal growth factor receptor 2 (HER2) expression level, which is the basis for selecting anti-HER2-targeted therapy. Anti-HER2-targeted drugs have revolutionized the treatment of HER2-positive GC and improved its outcome over the last decade. However, although HER2-positive patients account for only around 10% of all GC patients, it is necessary to develop novel molecular biomarkers to guide targeted treatments in GC.

Epithelial-mesenchymal transition (EMT) is a biological process allowing the epithelial cells to transform into mesenchymal cells, and EMT plays a role in physiological and pathological processes, which include embryonic evolution, wound healing, tumor cell metastasis, and drug resistance (5, 6). Different signaling pathways are involved in EMT: transforming growth factor-beta (TGF-β) signaling pathway, Hedgehog signaling pathway, Wnt/β-catenin signaling pathway, and Notch signaling pathway (7–9). The changes of molecular expression levels in these pathways could modulate the EMT-related transcription factors such as Snail, Twist, Slug, and Zeb, leading to an increased expression of mesenchymal cell markers (8). The EMT process could speed up the invasion, dissemination, and migration rates of cancer cells, which contributes to the rapid deterioration of disease and chemotherapeutic resistance. EMT markers were proven to be a critical prognosticator for different tumors, including glioma, endometrial cancer, and also GC (10–12).

Cancer stem cells (CSCs) are a small population of tumor cells playing a pivotal role in tumor progression, drug resistance, and survival of tumor cells. CSCs cause chemotherapeutic resistance and tumor recurrence through different mechanisms such as exporting cytotoxic drugs out of the cell through multidrug resistance (MDR) pumps, developing stronger DNA repair mechanisms, and reducing sensitivity to redox stress to prevent senescence (13–15). CSCs have been discovered to predict poor prognosis in many solid malignancies, including GCs, and inhibition of the CSC population may be an appropriate therapeutic strategy to prevent tumor recurrence and metastasis (16).

Literature surveys have revealed that there is an overlap between EMT stimuli and CSCs; activation of EMT-related transcription factors could increase the expression level of genes involved in prompting CSC transformation. Vesna et al. demonstrated that breast cancer cells would develop CSC phenotypes under the influence of TWIST overexpression (17). The tight connection between the EMT process and CSCs is observed in GC as well. Yoon et al. reported that activation of RTK-RAS signaling promoted EMT in GC cells, thus leading to the acquisition of CSC phenotypes (enrichment of CD44 expression) and invasive capabilities (18). There is mounting evidence suggesting that two processes may share common signaling pathways including TGF-β, Wnt/β-catenin, Hedgehog, Notch, and STAT3 (19). Considering the close relationship between these two processes in regulating each other and the common pathways they share, identifying molecular biomarkers related to both processes can achieve higher prognostic value and aid in the discovery of targeted treatment options.

Malta et al. used an innovative one-class logistic regression machine learning algorithm (OCLR) to calculate mRNA expression-based stemness index (mRNAsi), which indirectly reflected the activity of CSCs and the tumor differentiation state (20). Previous studies demonstrated that mRNAsi was a prognostic factor for GC (21). Therefore, it was reasonable to screen differentially expressed genes (DEGs) between high- and low-mRNAsi groups to identify stemness-related prognostic genes. The SERM signature for prognostic and drug sensitivity prediction was then developed by screening overlapped genes of CSC and EMT processes through statistical analyses, followed by the construction of a nomogram by integrating the signature and other clinical parameters.

## Materials and Methods

### Data Collection and Processing

RNA-sequencing matrix and clinical data of GC samples were downloaded from TCGA database (https://portal.gdc.cancer.gov/). “HT-Seq COUNT” and “HT-Seq FPKM” workflow types of TCGA stomach adenocarcinoma (TCGA-STAD) were downloaded, which included a total of 375 GC tissue samples and 32 adjacent normal samples. Clinical information was constituted by age, sex, TNM level, pathological stage, grade, survival time, and survival states. The mRNAsi of TCGA-STAD were obtained from Malta’s previous studies (20). Patients who met the following criteria were included in the subsequent analyses: (1) RNA-seq matrix sample ID name can be matched to mRNAsi ID name from the literature; (2) patients with completed clinical data for further analyses; and (3) clinical follow-up time no less than 30 days. Thus, 296 patients (296 tumor samples and corresponding mRNAsi level) were included in constructing CSC- and EMT-related prognostic gene signature (**Supplementary Table S1**). The microarray matrix and clinical data of GSE66229, GSE15459, and GSE26942 were downloaded from the GEO database (https://www.ncbi.nlm.nih.gov/geo/). We extracted the data of GC cell lines mRNA expression level and the information of different antineoplastic drugs IC50 of 32 GC cell lines from the CCLE database (https://sites.broadinstitute.org/ccle/).

### Identification of CSC- and EMT-Related Genes

Patients were categorized into low- and high-mRNAsi groups based on the median value of mRNAsi. The differentially expressed genes (DEGs) between low- and high-mRNAsi groups were screened using the “edgeR” R package with false discovery rate (FDR) <0.05 and |log_2_ fold change| >1. The heatmap and volcano plot were drawn by the R package “ggplot2” and “tinyarray” to visualized the differential analysis. The gene set HALLMARK_EPITHELIAL_MESENCHYMAL_TRANSITION was downloaded from the Molecular Signatures Database (MsigDB), which included the EMT-related genes for further analysis. To decipher gene signatures related to both CSCs and EMT, we screened overlapped genes between filtered DEGs and the EMT gene set. Based on the aforementioned strategies, 60 genes representing CSC and EMT crosstalk were finally identified.

### Construction of a 4-Gene-Based Prognostic Signature

A total number of 296 STAD patients with complete clinical data were enrolled in the construction of the CSC- and EMT-relevant prognostic signature. The univariate Cox regression analyses were conducted on CSC- and EMT-related genes and of which a *p*-value of less than 0.05 were considered the genes that significantly impact the survival of GC patients. The aforementioned genes were collected and pooled into the least absolute shrinkage and selection operator (LASSO) Cox regression algorithm, which minimized multicollinearity between different genes, to further reduce selected genes with the “survival” and “glmnet” R package. A risk prognosis model composed of 4 genes was established based on the linear combination of regression coefficients obtained from multivariate Cox regression analyses and gene expression values. The risk score of each patient was calculated by the formula that Risk score = sum of coefficients × gene expression level. The median value of risk score was used to separate samples into high- and low-risk groups in TCGA cohort. The same cutoff value was applied in the validation cohorts.

### Exploration of the Potential Biological Pathways for the Prognostic Signature in GC

To further explore the significant biological pathways potentially involved in the high-risk patients compared with low-risk patients, we conducted gene set enrichment analysis (GSEA) by “clusterProfiler” R package in the TCGA-STAD and GSE66229 cohorts between high- and low-risk patients. When adjust *p*-value <0.05 and FDR <0.25 after performing 1,000 permutations in GSEA analysis, gene sets were considered to be dramatically enriched. “Hallmark gene sets” were downloaded from the MsigDB for GSEA analysis. We then chose the upregulated genes in high-risk group to perform Kyoto Encyclopedia of Genes and Genomes (KEGG) enrichment analyses using “clusterProfiler” R package with a *p*-value <0.05 and a *q*-value <0.05.

To demonstrate the close relationship between the gene signature and EMT processes, we compared the risk score level among four GC molecular subtypes (MSS/TP53 activation, MSS/TP53 loss, MSI, and MSS/EMT) associated with distinct clinical outcomes (4). Furthermore, Pearson’s correlation analysis was performed between the risk score and the mRNA expression level of EMT markers (TWIST1, TWIST2, CDH2, FN1, SNAI1, SNAI2, MMP2, MMP9, ZEB1) (22).

### Assessment and Validation of the Prognostic Value of the Gene Signature on OS and DFS in the Public Database

Kaplan-Meier survival analyses and log-rank tests were implemented to evaluate the predictive value of the signature by using the R package “survival.” With “survival ROC” R package, time-dependent receiver operating characteristic (ROC) curves were conducted to determine the sensitivity and specificity of the risk score by measuring the area under the curve (AUC) (23). Univariate and multivariate Cox regression analyses of clinical characteristics and risk score were applied to evaluate whether the risk model was an independent prognostic factor for OS. The prognostic value of the established gene signature was validated in external validation cohorts GSE66229 and GSE15459.

Except for OS, DFS is also a crucial indicator for evaluating the disease progression, especially for the early stage of GC. The precise and accurate prediction of DFS could guide clinicians in formulating subsequent treatment plans. We wonder if the prognostic gene signature could be applied to predict the DFS of GC patients. In the GSE66229 cohort, survival curves between high- and low-risk groups of patients were depicted and compared using Kaplan-Meier estimates and log-rank test, respectively. Whether the risk score was an independent outcome predictor linked with DFS was determined by univariate and multivariate Cox analyses.

### Application of Quantitative Reverse Transcription Polymerase Chain Reaction in GC Cell Lines and Tissues

To further validate the prognostic value of gene signature, we collected 126 GC patients who were diagnosed between 2007 and 2011 with complete clinical information from Fudan University Shanghai Cancer Center. In these cases, the expression level of genes that were included in the CSC- and EMT-related prognostic signature was validated by quantitative reverse transcription polymerase chain reaction (RT-qPCR) in gastric cancer tissues of 126 patients. The clinical information and RT-qPCR results of patients in our cohort are presented in **Supplementary Table S2**. We also explored the mRNA expression levels of four genes in eight human GC cell lines (HGC-27, MKN-28, SGC-7901, BGC-823, MGC-803, AGS, NCI-N87, MKN45) and gastric mucosal cell line GES-1. Total RNAs were extracted from the GC cell lines and clinical tissue specimens using TRIzol reagent (Invitrogen, Waltham, MA, USA). First-strand cDNA was synthesized using the Evo M-MLV RT Premix kit for qPCR (Accurate Biology, Hunan, China) according to the manufacturer’s instructions. Relative RNA levels determined by RT-qPCR were measured on a 7900 Real-Time PCR System with the SDS 2.3 software sequence detection system (Applied Biosystems, Waltham, MA, USA) using the SYBR Green (Accurate Biology, Hunan, China) method. β-Actin was employed as the internal control to quantify the mRNA levels of model genes. The relative levels of RNA were calculated using the comparative CT(2−ΔΔCT) method. We listed the specific primers for SERPINE1, EDIL3, RGS4, MATN3, and β-actin in **Supplementary Table S3**.

### Clinical Subgroup Analysis of the Prognostic Signature

To investigate whether the prognostic gene signature had the predictive power for OS and DFS in subgroups of patients with different clinical characteristics, patients were divided into subgroups based on age, gender, T stage, N stage, and TNM stage. The *p*-value of the log-rank test obtained by comparing survival outcomes between different risk levels of patients was used to measure the prognostic value of gene signature in each clinical subgroup.

### Construction and Assessment of the Signature-Based Nomogram

A nomogram including age, gender, pathological parameters, and risk score was constructed by “rms” R package to predict the 1-, 3-, and 5-year OS of GC patients. The concordance index (C-index) and AUC of the nomogram were calculated by “rms” R package to reflect the discrimination ability of the model. The concordance between the predicted outcome and actual survival outcome was reflected by plotting the nomogram calibration curves. Decision curve analyses (DCA) were conducted to evaluate the net benefit of nomogram at different threshold values compared with other simple or complex models by “ggDCA” R packages (24). We applied the same methods to validate the accuracy of nomogram in the external validation cohort GSE66229.

### Assessment of the Gene Signature Prognostic Value on Antineoplastic Drug Sensitivity

Research advances have provided solid evidence for the contribution of EMT and CSC activation to primary and developed chemotherapeutic drug resistance (25). Therefore, we speculated that the gene signature we developed, which was related to CSC and EMT process, could predict the GC patients’ susceptibility to chemotherapeutic drug treatment. We downloaded the data of GC cell line mRNA expression level and the information of different antineoplastic drugs IC50 of GC cell lines from the CCLE database. The risk score of each GC cell line was calculated by the formula we developed. The relationship between IC50 of drugs and risk score was then analyzed by conducting Pearson’s correlation analyses. The GSE26942 cohort, which was based on GPL6947 (Illumina HumanHT-12 V3.0 expression beadchip) contained 202 GC patients’ samples, including 106 patients treated with adjuvant chemotherapy and 96 patients untreated after surgery. To analyze the capacity of the signature on predicting chemotherapeutic drug sensitivity, we chose the patients who accepted the adjuvant chemotherapy for further analysis. The previous formula was used to compute each patient’s risk score, and patients were divided into high- and low-risk groups based on the same cutoff applied in TCGA cohort. To test the prognostic value of gene signature on chemotherapeutic drug susceptibility, we applied Kaplan-Meier analyses between high- and low-risk groups. We conducted multivariate Cox regression analyses of clinical characteristics and risk score to assess whether the gene signature was an independent prognostic factor for drug sensitivity.

### Statistical Analyses

R 4.1.0 software (https://www.R-project.org) and GraphPad Prism 7 were used for statistical analysis and graphing in this article. Wilcoxon test and Kruskal-Wallis were used for risk score comparisons between EMT and non-EMT groups. Pearson’s correlation analysis was implemented to analyze the correlation between risk score and EMT markers and calculate the correlation coefficient. Kaplan-Meier curve analysis with log-rank test was conducted to compare survival differences between different groups of patients. Statistical significance was considered *p* < 0.05, and all *p*-values were two tailed.

## Results

### Construction of the SERM Prognostic Signature

**Figure 1:**
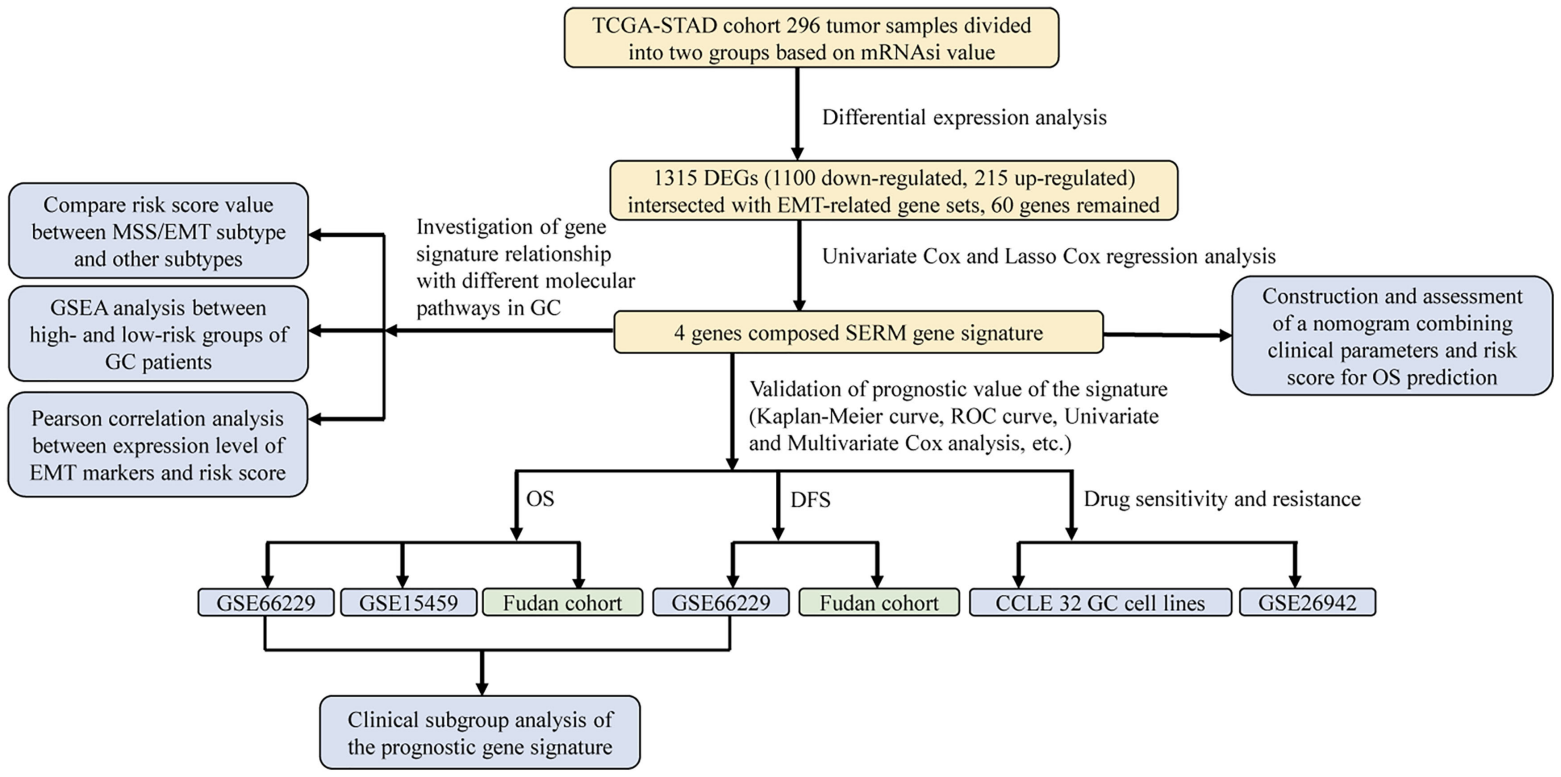
The flowchart presenting the procedure and processes of our study.

**Figure 2:**
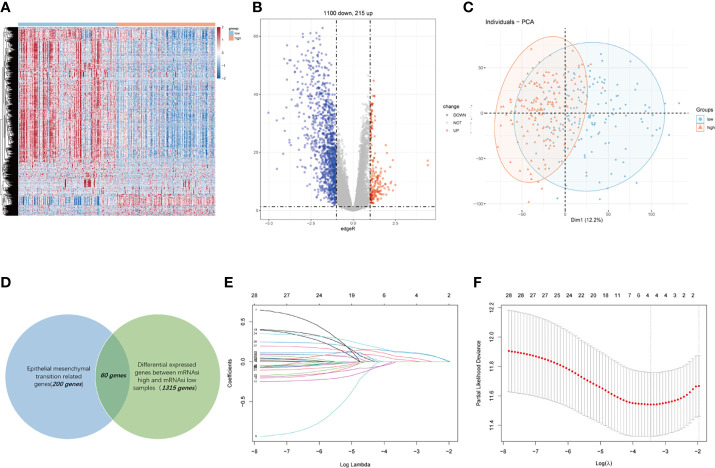
Construction of a four-gene-based SERM prognostic signature for GC. **(A, B)** Heatmap **(A)** and volcano plot **(B)** reflected DEGs between high- and low-mRNAsi groups. **(C)** PCA plot presented the differences in overall gene expression level between high- and low-mRNAsi group. **(D)** Venn diagram indicated the overlapped genes between DEGs and EMT gene set. **(E)** LASSO coefficient profiles of 29 prognostic genes. **(F)** Ten-fold crossvalidation for tuning parameter selection in the LASSO model.

We conducted this study methodically based on the steps presented in the flow chart (**Figure 1**). We screened 1,315 DEGs (1,100 downregulated, 215 upregulated) between high- and low-mRNAsi group; heatmap reflecting differential gene expression patterns and volcano plot which directly identify significantly differentially expressed genes among two groups were exhibited in **Figure 2A, B**. PCA plot presented the differences in gene expression between high- and low-mRNAsi groups (**Figure 2C**). The Venn diagram indicated the overlapped genes between DEGs and EMT gene set (**Figure 2D**). Univariate Cox regression was applied on overlapped genes with *p*-value less than 0.05 (**Supplementary Table S4**), after which, 29 genes were subjected to LASSO Cox regression analyses to construct a prognostic signature based on CSC and EMT processes for evaluating the prognosis of GC patients (**Figures 2E, F**). Ultimately, a prognostic gene signature including SERPINE1, EDIL3, RGS4, and MATN3 four genes (SERM signature) was constructed. The formula of calculating prognostic risk score could be indicated as: 0.211372 × (expression level of SERPINE1) + 0.103095 × (expression level of EDIL3) + 0.071508 × (expression level of RGS4) + 0.210286 × (expression level of MATN3). The coefficients of the four genes in the prognostic model were all greater than zero, which indicated that they were all predictors of poor prognosis. GC patients in the TCGA-STAD cohort were split into high- and low-risk groups based on the median risk score which was identified as −0.018421. Patients in validation cohorts were separated into two groups based on the same cutoff value.

### Enrichment and Statistical Analysis Revealed the Potential Mechanisms of the SERM Signature

**Figure 3:**
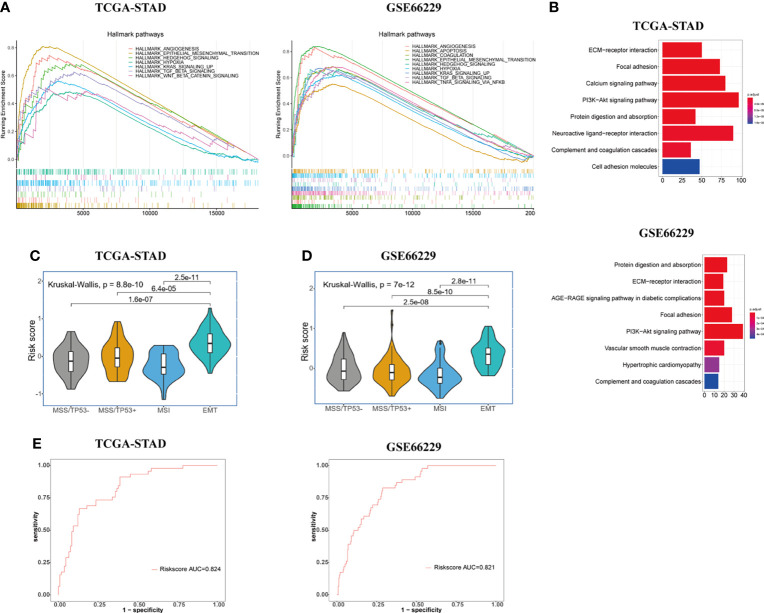
Exploration of the potential biological pathways for the prognostic signature in GC. **(A)** GSEA analysis was carried out to investigate the enrichment score and *p*-value of hallmark gene sets between the high- and low-risk groups in TCGA-STAD and GSE66229 cohorts. **(B)** KEGG enrichment analysis for upregulated genes in the high-risk group. **(C, D)** Risk score value were evaluated among four molecular subtypes (MSS/TP53 activation, MSS/TP53 loss, MSI, and MSS/EMT) in both TCGA-STAD **(C)** and GSE66229 **(D)** cohorts. **(E)** The ROC curves were used to testify the ability of risk score level in discriminating EMT and non-EMT molecular subtypes in the TCGA-STAD and GSE66229 cohorts.

We obtained ranked gene lists between high- and low-risk groups by using “limma” R package and then conducted GSEA analysis using “hallmark gene sets” downloaded from MsigDB. GSEA analysis indicated that in addition to powerful activation of EMT process, the enrichment of angiogenesis process, hypoxia, TGF-β pathway, Hedgehog pathway, and KRAS signaling pathways was observed in the high-risk group of GC patients in both TCGA and GSE66229 cohorts (**Figure 3A**). Accumulating evidence indicates that TGF-β and Hedgehog pathways play significant roles in EMT process and formation of CSCs, suggesting that the signature has a strong association with both processes (19, 26, 27). What is more, KRAS signaling pathway, hypoxia, and angiogenesis processes were demonstrated to accelerate tumorigenesis and metastasis, thus leading to disease progression and poor prognosis in GC (18, 28, 29). The results of KEGG enrichment analyses for gene signature are shown in **Figure 3B**. Protein digestion and absorption, focal adhesion, ECM-receptor interaction, complement, coagulation cascades, and PI3K-Akt pathways were five top significant KEGG pathways related to high-risk group in both cohorts.

To further elucidate and demonstrate the close relationship between the gene signature and EMT process, we performed a Kruskal-Wallis test to investigate the differential risk score value among four molecular subtypes (MSS/TP53 activation, MSS/TP53 loss, MSI, and MSS/EMT) proposed by the ACRG group in TCGA-STAD cohort. Wilcoxon test was used to compare the risk score value between MSS/EMT group and other subtypes. In the TCGA-STAD cohort, the risk score level was significantly higher in the MSS/EMT group compared with MSS/TP53 activation, MSS/TP53 loss, and MSI subtypes (*p* < 0.05) (**Figure 3C**). To make the results more reliable, we applied the same analyses in the GSE66229 cohort and the results turned out to be identical with TCGA cohort (**Figure 3D**). Furthermore, we conducted ROC analysis to further elucidate the remarkably differential risk score value between EMT and non-EMT subtypes. The result of ROC analysis illustrated that the established gene signature based on CSC and EMT processes showed good discriminatory ability between EMT and non-EMT subtypes (**Figure 3E**). Additionally, we computed the correlation index between the mRNA expression level of EMT markers and risk score value in the TCGA and GSE66229 cohorts using “ggpubr” R package by Pearson’s correlation analysis. Recent studies have shown the signaling pathways involved in the EMT process change the gene expression through modulating the transcription factors such as Snail, Twist, and ZEB (8). The changes of EMT-related transcription factors could increase the expression level of mesenchymal cell markers and matrix metalloproteinases (MMP), especially MMP-2 and MMP-9 (30). Therefore, We chose EMT-related transcription factors and their downstream proteins as EMT markers in our study. The results were presented in **Supplementary Figure S1**. We could conclude that the mRNA expression level of all EMT markers we investigated in our study showed obvious positive correlations with risk score (*p* < 0.001).

### Assessment and Validation of the Prognostic Value of SERM Signature on OS and DFS in the Public Database

**Figure 4:**
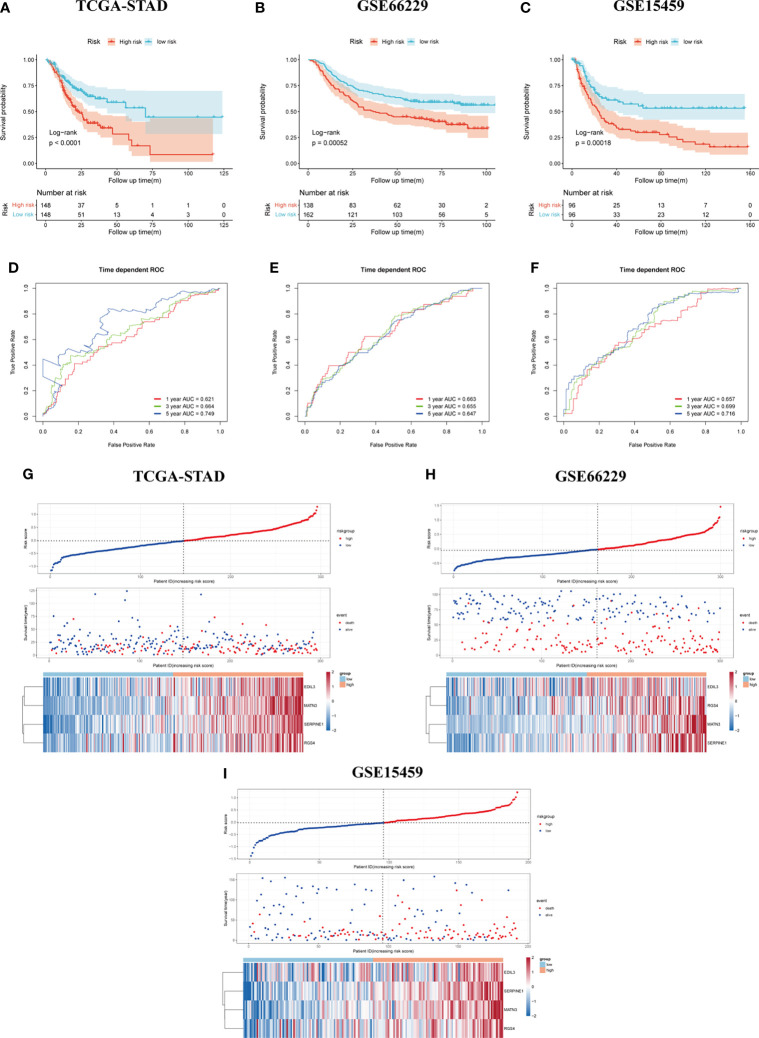
Assessment and validation of the SERM prognositc signature on OS prediction. **(A–C)** Kaplan-Meier analysis of OS between the high- and low-risk groups based on the SERM signature in the TCGA-STAD **(A)**, GSE66229 **(B)**, and GSE15459 **(C)** cohorts. **(D–F)** Time-dependent ROC curves of the SERM signature in the TCGA-STAD **(D)**, GSE66229 **(E)**, and GSE15459 **(F)** cohorts. **(G–I)** The distributions of risk score, survival status, and heatmap of the SERM signature expression levels in the TCGA-STAD **(G)**, GSE66229 **(H)**, and GSE15459 **(I)** cohorts.

The result of survival analysis between high- and low-risk groups is presented in **Figure 4A**, the high-risk group exhibited significantly shorter OS compared with the low-risk group (*p* < 0.0001). The predictive value of the four-gene-based model was evaluated by calculating the AUC value under the time-dependent ROC curve. The 1-, 3-, and 5-year AUCs were 0.621, 0.664, and 0.749, respectively (**Figure 4D**). To validate the prognostic ability of gene signature, the risk score was calculated by the same formula in the GSE66229 and GSE15459 cohorts. To divide patients into different groups, the same cutoff of risk score was used in two validation cohorts. In the GSE66229 cohort, 162 patients belonged to the low-risk group and the remaining 138 patients were categorized as high-risk group. Two groups owned the same number of patients in the GSE15459 cohort, which meant 96 patients were included in each group. The difference in survival time between high- and low-risk groups was also statistically significant in two validation cohorts (**Figures 4B, C**). In the GSE66229 cohort, the AUCs of 1-, 3-, and 5-year ROC were 0.663, 0.655, and 0.647, respectively. The 1-, 3-, and 5-year AUCs were 0.657, 0.699, and 0.716 in GSE15459, showing a good prognostic discrimination of the SERM signature (**Figures 4E, F**). Univariate Cox regression analysis of risk score showed that it was an adverse prognostic factor for GC. Furthermore, multivariate Cox analysis in three cohorts indicated that the risk score was an independent prognostic factor for GC patient OS (**Supplementary Tables S5**-**S7**). These results demonstrated that the gene signature we derived by LASSO Cox regression for OS prediction could be used as a valuable prognostic marker. The distributions of risk score, survival status, and heatmap of gene signature expression levels of the TCGA-STAD, GSE66229, and GSE15459 cohorts are shown in **Figures 4G–I**.

We tested the prognostic value of SERM signature on DFS of GC patients in the GSE66229 cohort. The same cutoff value (−0.018421) was used for dividing GC patients in the GSE66229 cohort into high- and low-risk groups to analyze the survival differences between two groups by Kaplan-Meier plot with the log-rank test. The result is shown in **Supplementary Figure S2A**, which suggested the SERM signature owning a strong predictive power for DFS of GC patients who undergo a radical operation. We then performed time-dependent ROC analysis to evaluate the prognostic accuracy of the model and observed the values of 1-, 3-, and 5-year AUC were 0.653, 0.66, 0.695, respectively (**Supplementary Figure S2B**). Multivariate Cox regression indicated the risk score was an independent prognostic factor for predicting DFS of GC patients (**Supplementary Figure S2C**). The distributions of risk score, survival status, and heatmap of gene signature expression levels of the GSE66229 cohort are shown in **Supplementary Figure S2D**.

### RT-qPCR Validation of SERM Signature Gene Expression in Both GC Cell Lines and the Fudan Cohort

**Figure 5:**
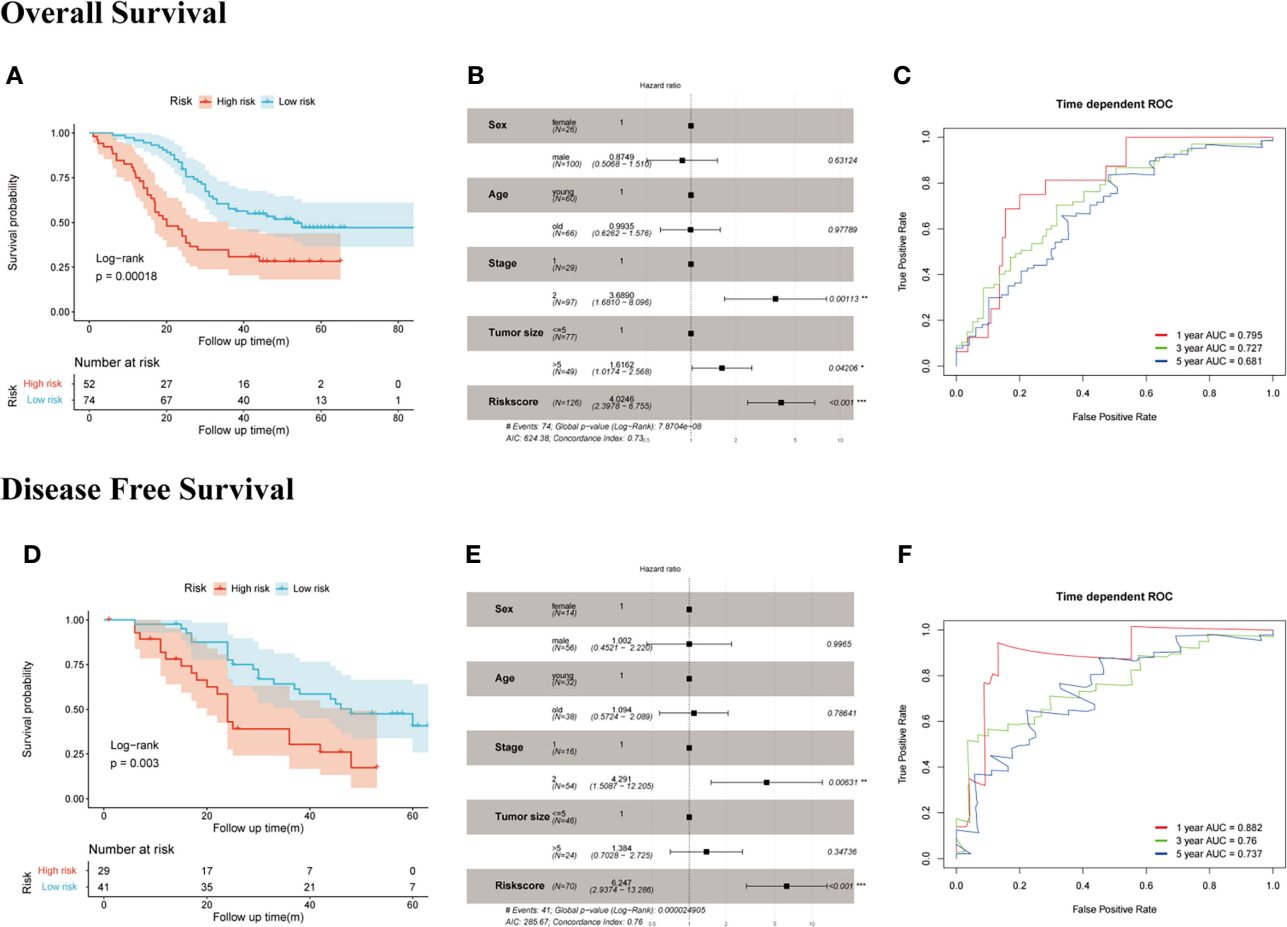
Evaluation of the prognostic value of SERM signature on OS and DFS prediction in Fudan cohort. OS: **(A)** Kaplan-Meier analysis of OS according to risk score value. **(B)** Multivariate Cox regression analysis of clinical parameters and risk score. **(C)** Time-dependent ROC curves of the SERM signature for 1-, 3-, and 5-year OS prediction. DFS: **(D)** Kaplan-Meier analysis of DFS according to risk score value. **(E)** Multivariate Cox regression analysis of clinical parameters and risk score. **(F)** Time-dependent ROC curves of the SERM signature for 1-, 3-, and 5-year DFS prediction.

We measured the mRNA expression level of EDIL3, SERPINE1, RGS4, and MATN3 with RT-qPCR in GC cell lines and Fudan cohort. The results for members of SERM signature gene expression level in different GC cell lines are presented in **Supplementary Figure S3**, which to some extent would provide guidance for us to choose GC cell lines for underlying molecular biological mechanism detection in further study. To validate the prognostic ability of gene signature on OS prediction, the risk score was calculated for each patient in our cohort according to the formula and coefficient obtained from multivariate Cox regression analysis in the TCGA cohort. We applied the same cutoff of the risk score in our validation cohort. In total, 52 and 74 patients were divided into high- and low-risk groups, respectively. The Kaplan-Meier analysis between the two groups demonstrated that compared with the low-risk group, the OS in the high-risk group was significantly poorer (**Figure 5A**). Univariate Cox regression analysis indicated risk score was an important marker influencing the OS of GC patients (*p* < 0.001). Multivariate Cox regression analysis based on risk score with other clinical parameters suggested risk score was an independent poor prognostic marker for OS **(****Figure 5B**). The prognostic value of the risk model on DFS prediction in Fudan cohort was also analyzed. The risk score was computed for each patient in our cohort with the formula and coefficient obtained from TCGA cohort. Patients were separated into high- and low-risk groups according to the same cutoff and each group owned 29 and 41 patients, respectively. Furthermore, we conducted the Kaplan-Meier analysis between two groups, and the results indicated the DFS in the high-risk group was significantly poorer compared with the low-risk group (**Figure 5D**). The *p*-values of the risk score of univariate and multivariate Cox analyses were both lower than 0.001, indicating it was an independent prognostic marker for DFS (**Figure 5E**). Time-dependent ROC analysis suggested the signature’s good performance on predicting OS and DFS in GC patients (**Figures 5C, F**).

### Assessment of the Prediction Power of the SERM Prognostic Signature in Clinical Subgroups by Kaplan-Meier Plot

**Figure 6:**
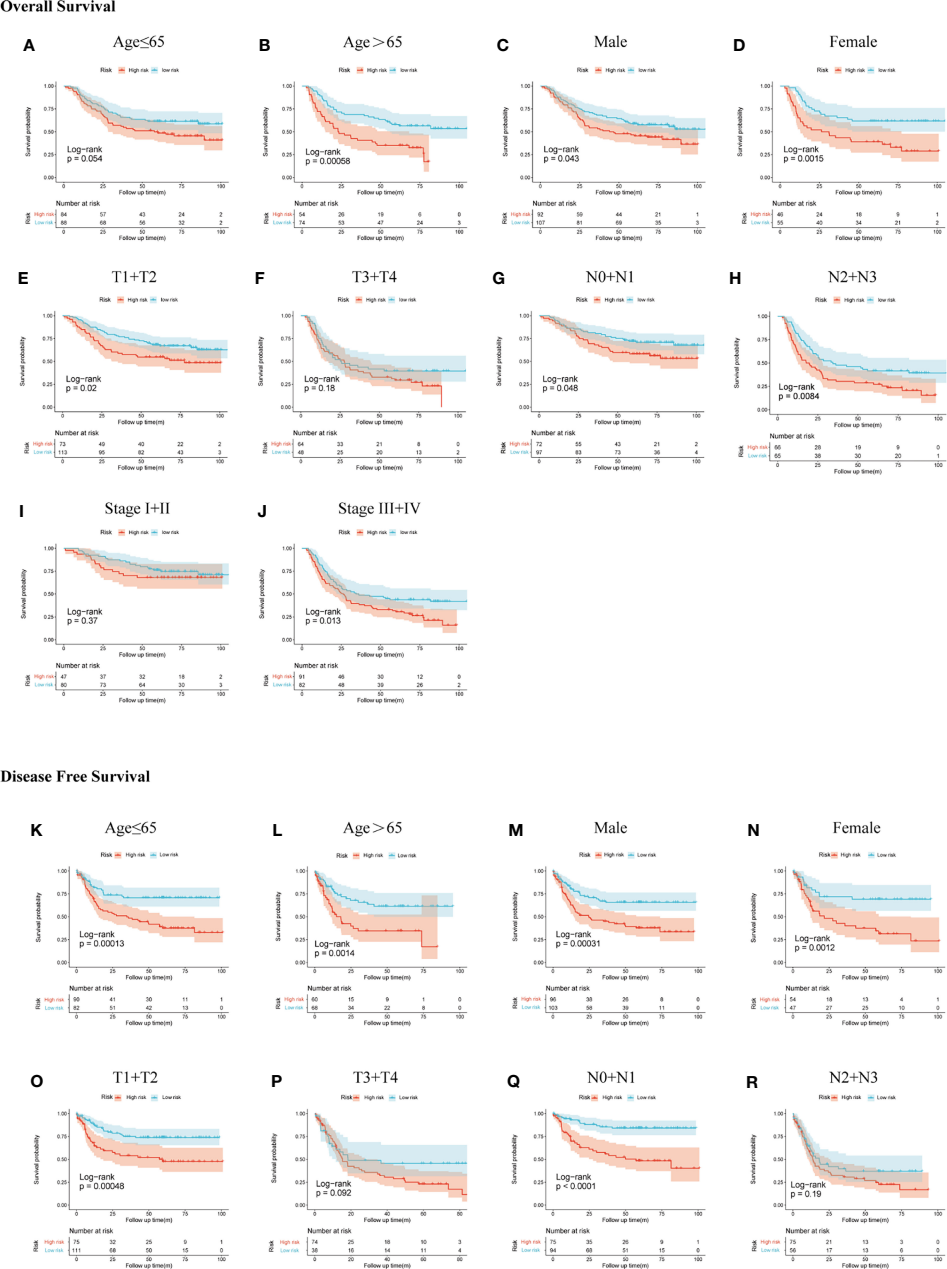
Subgroup analysis of OS and DFS in the GSE66229 cohort. OS Kaplan-Meier plot: **(A)** age ≤65, **(B)** age >65, **(C)** male, **(D)** female, **(E)** T1 + T2, **(F)** T3 + T4, **(G)** N0 + N1, **(H)** N2 + N3, **(I)** stage I + II, and **(J)** stage III + IV; DFS Kaplan-Meier plot: **(K)** age ≤65, **(L)** age >65, **(M)** male, **(N)** female, **(O)** T1 + T2, **(P)** T3 + T4, **(Q)** N0 + N1, and **(R)** N2 + N3.

We demonstrated the predictive power of gene signature in different clinical subgroups based on age, gender, T stage, N stage, and TNM stage in the GSE66229 cohort. According to the previous risk score cutoff, patients in subgroups were split into high- and low-risk groups, and the Kaplan-Meier analyses were conducted to detect if the signature could be used as a prognostic indicator for OS and DFS in clinical subgroups. As shown in **Figures 6A–J**, patients with high risk had a worse prognosis of OS than patients with low risk in patients >65 years (*p* < 0.001), female (*p* = 0.0015), male (*p* = 0.043), T1-2 (*p* = 0.02), N0-1 (*p* = 0.048), N2-3 (*p* = 0.0084), stage III-IV (*p* = 0.013) subgroups; however, the prognostic signature was incompetent in distinguishing the OS of the high risk from the low-risk group in age ≤65 years (*p* = 0.054), T3-4 (*p* = 0.18), and stages I–II (*p* = 0.37) subgroups. In **Figures 6K–R**, patients with high risk had a worse prognosis of DFS than patients with low risk in patients >65 years (*p* = 0.0014), ≤65 years (*p* < 0.001), male (*p* < 0.001), female (*p* = 0.0012), T1-2 (*p* < 0.001), N0-1 (*p* < 0.001), stages I–II (*p* = 0.011), and stages III–IV (*p* = 0.0098). While in T3-4 and N2-3 subgroups, *p*-values of the Kaplan-Meier plot were 0.092 and 0.19, respectively.

### Construction and Validation of the SERM Signature-Based Nomogram for OS Prediction in GC

**Figure 7:**
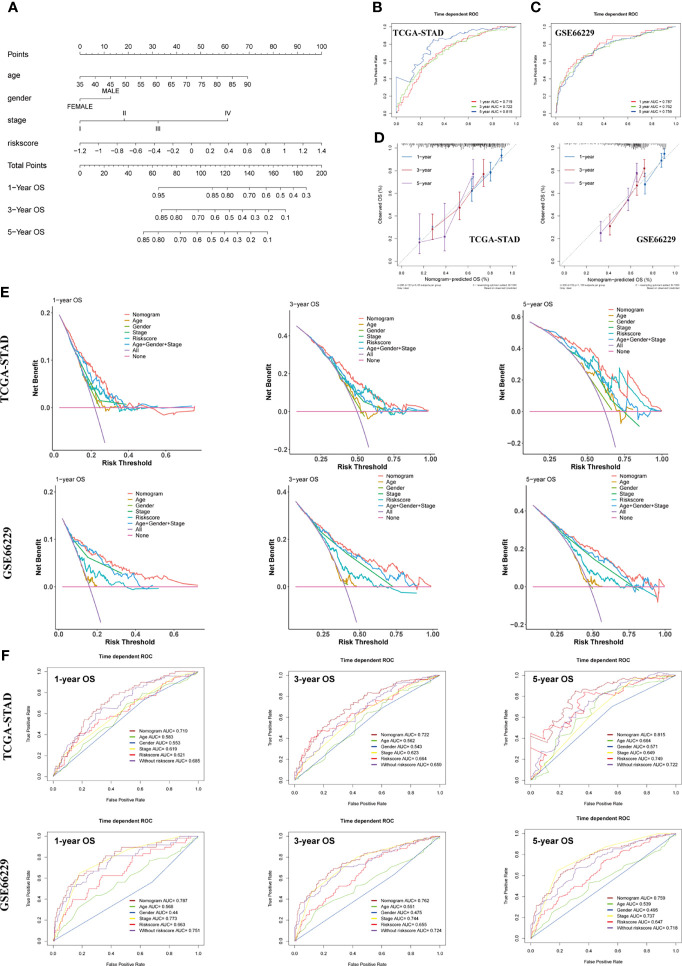
Construction and assessment of a nomogram for OS prediction. **(A)** A nomogram integrating age, gender, TNM stage, and risk score was constructed in the TCGA-STAD cohort. **(B, C)** Time-dependent ROC curves of the nomogram for 1-, 3-, and 5-year OS prediction in the TCGA-STAD **(B)** and GSE66229 cohorts **(C)**. **(D)** Calibration curves of the nomogram for 1-, 3-, and 5-year OS prediction in the TCGA-STAD and GSE66229 cohorts. **(E)** DCA curves were performed to evaluate the net benefit of the nomogram, age, gender, pathological characteristics, and risk score in the TCGA-STAD and GSE66229 cohorts. **(F)** Time-dependent ROC curves of the nomogram, age, gender, TNM stage, risk score, and model without risk score for 1-, 3-, and 5-year OS prediction in the TCGA-STAD and GSE66229 cohorts.

To make the model more applicable in clinical use, we next established a nomogram, which integrates age, gender, TNM stage, and risk score to achieve the purpose of optimizing current indicators for long-term OS prediction by multivariate Cox regression in TCGA-STAD cohort (**Figure 7A**). The validation cohort GSE66229 was used to test the predictive accuracy of the nomogram. The nomogram-combined clinical characteristics and the SERM signature were used to predict 1-, 3-, and 5-year survival probabilities. Each patient would get a unique score based on the constructed nomogram, and the higher the score was, the worse the prognosis. The discrimination degree, concordance, and clinical usefulness of the nomogram were quantified by time-dependent ROC curve, nomogram calibration curve, and DCA.

For the constructed nomogram in the training cohort, the C-index of the nomogram for survival prediction was 0.70 and the AUCs of 1-, 3- and 5-year ROC were 0.719, 0.722 and 0.815, respectively (**Figure 7B**). The C-index of nomogram built with age, gender, TNM stage, and risk score in the GSE66229 cohort was 0.72. The AUC values of ROC were 0.787 at 1 year, 0.762 at 3 years, and 0.759 at 5 years (**Figure 7C**). The nomogram calibration curves of training and validation cohorts presented in **Figure 7D** exhibited a good consistency between nomogram-predicted OS and the actual observation at 1-, 3-, and 5-year OS, which further demonstrated the accuracy of the nomogram. Shown by the DCA curve in **Figure 7E**, the nomogram yielded a better net benefit compared with individual predictive factors and the model without risk score, illustrating the combined nomogram could give guidance to clinicians to make a better prediction on patient OS prognosis. Compared with age, gender, TNM stage, and model without risk score, the combined nomogram exhibited the largest AUC for 1-, 3-, and 5-year OS prediction in both training and validation cohort, suggesting integrating risk score into Cox model could improve the discrimination capacity of the model (**Figure 7F**).

### The Developed SERM Signature Could Predict Antineoplastic Drug Sensitivity in GC Cell Lines and GC Patients

**Figure 8:**
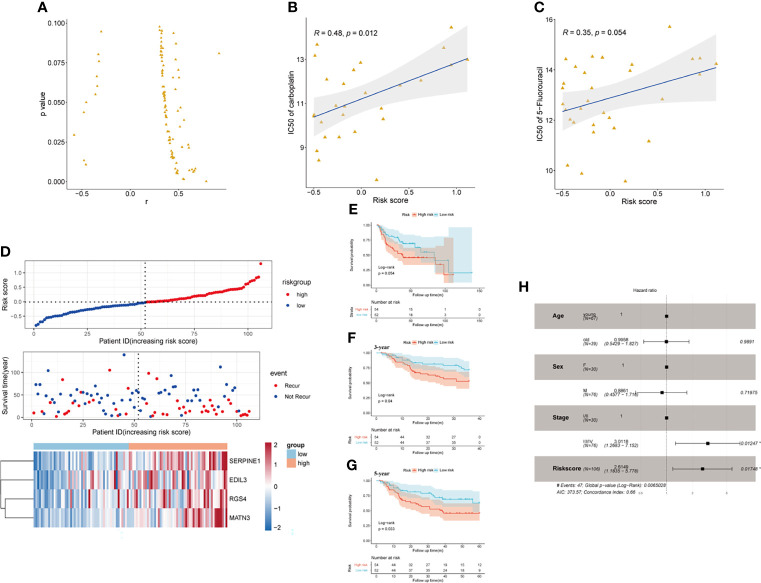
The prognostic value of the signature on drug sensitivity in GC cell lines and GSE26942. **(A)** Risk score was positively related to IC50 of most antineoplastic drugs in GC cell lines. **(B, C)** Risk score was positively related to IC50 of carboplatin **(B)** and 5-flurouracil **(C)**. **(D)** The distributions of risk score, survival status, and heatmap of CSCs and EMT-related gene signature expression levels in GSE26942. **(E)** Kaplan-Meier analysis of DFS for patients who received adjuvant chemotherapy between high- and low-risk groups in GSE26942 cohort. **(F, G)** Kaplan-Meier analysis for 3- **(F)** and 5- year **(G)** DFS prediction between high- and low-risk groups in patients received adjuvant chemotherapy. **(H)** Multivariate Cox regression analysis of clinical parameters and risk score in the GSE26942 cohort.

Pieces of evidence have demonstrated that phenotypical changes associated with EMT process and stem cell characteristics lead to a reduced response of GC to chemotherapy. The upregulation of EMT markers (vimentin and N-cadherin), which may be regulated by the activation of TGF-β pathway, was reported to cause a worse response of GC to 5-FU (31). CD133 has been identified as a significant marker of CSCs in various cancers, including GC. GC patients with a high expression level of CD133 treated with an adjuvant cisplatin/5-FU therapy had shorter OS and DFS than those CD133-low patients, which indicated that CD133 seemed to contribute to chemoresistance in GC (32). Thus, we reasonably speculated that the established SERM signature could predict GC patients’ susceptibility to chemotherapeutic drugs. To confirm our conjecture, we analyzed the prognostic value of gene signature on drug sensitivity in both GC cell lines and patients. Pearson’s correlation tests were used to assess the relationship between antineoplastic drugs IC50 and riskscore of 32 GC cell lines. We screened the results of Pearson’s correlation test with a *p*-value <0.1; the results showed that the risk score was positively related to IC50 of most antineoplastic drugs, which indicated that the higher level of risk score was associated with drug resistance (**Figure 8A**). As shown by **Figures 8B, C**, IC50 of carboplatin and 5-FU chemotherapeutic drug was positively correlated with the risk score value. Additionally, We chose 106 patients who were treated with adjuvant chemotherapy in GSE26942 cohort to assess the capacity of the signature on predicting chemotherapeutic drug sensitivity. We selected DFS as an indicator reflecting patients’ response to adjuvant chemotherapy. The distributions of risk score, survival status, and heatmap of gene signature expression levels of the GSE26942 cohort were shown in **Figure 8D**. The Kaplan-Meier plot illustrated that the patients with high risk score had shorter DFS compared with patients with low-risk score (*p* = 0.054) (**Figure 8E**). The results of Kaplan-Meier analysis for 3- and 5-year DFS between high- and low-risk groups are presented in **Figure 8F** (*p* = 0.040) and **Figure 8G** (*p* = 0.033), respectively. The result of univariate (*p* = 0.012) and multivariate Cox analyses suggested the signature was an independent prognostic factor for predicting GC patients’ susceptibility to adjuvant chemotherapy (**Figure 8H**).

## Discussion

The traditional prognostic systems such as TNM staging systems could be inaccurate under some conditions for predicting GC patients’ survival, so exploring specific and sensitive markers for survival prediction of GC patients remains an exigency. Evidence shows that EMT is closely related to the function of CSCs in addition to playing an important role in the metastasis of various tumors. Moreover, under the influence of the EMT process, tumor cells may acquire cancer stem-like properties, which leads to drug resistance, increased relapse, and metastasis in multiple kinds of tumors (26, 33). In addition, accumulating evidence verifies that there is an overlap between pathways mediating two processes, including TGF-β, Hedgehog, Wnt/β catenin, and Notch pathways (19). Choi et al. reported that the expression level of EMT markers (E-cadherin, β-catenin, Snail, vimentin) was correlated with stem cell marker expression level (34). CSC and EMT processes were proven to cause disease progression, poor prognosis, and drug resistance in lung cancer, esophageal cancer, breast cancer, and also GC (35–38). Thus, considering the common pathways and mechanisms two processes share and the same clinical impact they have, identification of transcriptional markers for these two processes will achieve a better prognosis and may give guidance to targeted therapies in GC patients.

In this study, we screened out CSC- and EMT-related mRNAs and identified a novel four-gene-based SERM signature and validated the prognostic value of signature in both public and Fudan cohorts. We calculated the risk score of patients using the formula we mentioned in the *Results* part. Patients were then divided into high- and low-risk groups based on the median value of risk score. The results of Kaplan-Meier survival analyses and time-dependent ROC curves in different cohorts indicated that the signature could effectively predict the survival and drug sensitivity to adjuvant chemotherapy of GC patients. To improve the clinical use of the gene signature, we combined clinical parameters with the risk score and build a nomogram to predict each GC patient’s OS in 1, 3, and 5 years. Discrimination degree, concordance, and clinical usefulness of nomogram were evaluated in TCGA and GEO cohorts, the results of which suggest its potential application values in patient’s risk stratification.

Four members in the signature were related to adverse clinical outcomes of GC patients, and they all have been reported as EMT-related negative predictors in various kinds of tumors. GSEA results in our study indicated that four members involved in the gene signature may have a crucial role in regulating TGF-β, Hedgehog, and Wnt pathways to promote the formation of CSCs through the EMT process in GC patients. Except for the mentioned pathways, we noticed the enrichment of angiogenesis process, hypoxia, and KRAS signaling pathways in the high-risk group. Literature demonstrated the compact association between these pathways and EMT or CSC-related processes. Twist1 was a transcription factor playing a crucial role in EMT and cancer stemness; Chen et al. indicated that in addition to traditional angiogenesis, the activation of the Twist-Jagged1-KLF4 axis could induce tumor-associated endothelial differentiation (39, 40). Previous literature indicated that the concurrent activated KRAS and depletion of p53 could reprogram EMT-like phenotypes and increase the expression of cancer stemness genes including CD133, EpCAM, and CD24 in prostate cancer (41). Changhwan et al. demonstrated the RTK-RAS signaling could enhance the activation of EMT signal and promote the expression of stemness-related transcription factors in human tumor-derived GC cells (18). Of note, hypoxia-inducible factor-1α (HIF-1α) were proven to regulate expression of EMT markers and EMT transcriptional factors (42). Recent studies conducted by Komal et al. discovered CSC accumulation in hypoxic niches and the anoxic conditions promoted the self-renewal ability of CSCs (43). We observed the upregulation of PI3K-Akt pathway-related genes in KEGG enrichment analysis, and previous studies indicated that this pathway was involved in both CSC and EMT processes (44). The results of GSEA and KEGG enrichment analyses revealed the potential molecular mechanisms of the gene signature, which might give guidance to the development of targeted therapy. SERPINE1 gene encodes a protein called plasminogen activator inhibitor-1 (PAI-1), which is a key regulator of the urokinase-type plasminogen activator (uPA) system. Previous reports demonstrated that the upregulation of SERPINE1 in breast cancer and pancreatic cancer tissue could be induced by TGF-β pathway activation (45–47). However, the pathway influencing SERPINE1 expression level in GC needs more investigation. Bhat-Nakshatri et al. discovered all-trans retinoic acid (ATRA) reduced the mammosphere-forming ability of cell lines by reducing the expression level of SERPINE1 in CSCs, suggesting SERPINE1 may be a pivotal molecule related to CSC formation (48). Increasing sherds of evidences have revealed that SERPINE1 was significantly upregulated in GC tissues compared with normal tissues and could lead to a poor prognosis. McCann et al. pointed out the poor prognosis caused by overexpression of SERPINE1 was related to the imbalance between fibrin deposition and fibrin degradation, inhibiting PAI-1 expression with miR-30c imitated enhanced plasmin activity by fibrin zymograms (49). EDIL3 is an extracellular matrix protein containing three EGF-like domains and the second domain could allow the interaction of EDIL3 with integrins. EDIL3 acts as a proangiogenic factor, a mediator of angiogenesis, and a regulator of endothelial cell adhesion and migration (50–52). The overexpression of EDIL3 was observed in several tumor types, including breast, bladder, liver, and lung carcinomas, and it associates with drug resistance and poor prognosis (53–56). Overexpression of EDIL3 in hepatocellular carcinoma could induce the phosphorylation of SRC, ERK, and SMAD2, leading to the activation of ERK and TGF-β signaling. The activation of these pathways could increase the transcription efficiency of mesenchymal markers and integrins, resulting in cell acquisition of the molecular and morphologic changes of CSCs and EMT (57). RGS4, is a kind of regulator of G-protein signaling (RGS) proteins that catalyze the dephosphorylation of guanosine triphosphate into guanosine diphosphate. Guda et al. found that silencing RGS4 in glioma cancer stem cells (GSCs) decreased the expression, secretion, and activity of MMP2, suggesting decreased invasive and migratory abilities of GSCs (58). However, Cheng et al. suggested that overexpression of RGS4 in NSCLC cells inhibits MM2/9 expression, thus leading to decreased invasion and migration (59). It was verified that RGS4 was upregulated in mesenchymal stem cells compared with diffuse-type GC, which may suggest that increased expression level of RGS4 may lead to cell EMT transition (60). The prognostic value of RGS4 in GC is not yet clear. MATN3 is a protein-coding gene encoding a member of von Willebrand factor A domain containing protein family, which is involved in the formation of filamentous networks in the extracellular matrix (61). Wu et al. performed bioinformatics and immunohistochemistry to prove that compared with a normal control group, MATN3 protein expression level was significantly higher in the GC tissue group. Furthermore, they found MATN3 was an independent factor to predict unfavorable prognosis in GC patients (62).

Although the prognostic signature was tested and validated in several different cohorts and the results turned out to be stable, our study still has some limitations. Firstly, the TNM stages recorded in TCGA and GEO cohorts were not computed according to the latest edition of AJCC staging system. It was difficult for us to unify the standard of the TNM stages because of the insufficient data recording. Secondly, although we developed a prognostic gene signature related to both CSC and EMT processes and demonstrated its accuracy, scientists should carry out more research on how these genes influence both pathways and how they are connected in GC. A legible understanding of biological mechanisms can give better guidance for clinical use. Thirdly, the GSE26942 cohort we used to demonstrate the prognostic value of gene signature on drug sensitivity and resistance only contained the microarray data before adjuvant chemotherapy treatment, so the change of gene expression level after treatment remained unknown. Therefore, it was difficult for us to analyze the relationship between the signature and developed drug resistance. Furthermore, the prognostic effect of the gene signature on chemotherapeutic resistance for advanced GC patients who received palliative chemotherapy needs more exploration. Of note, GC patients always received combination chemotherapy, or sometimes with targeted agents, so the mechanism of chemoresistance will be quite complicated and elusive. Consequently, we should attach more importance to the exploration of basic biological mechanisms for chemotherapeutic resistance in GC. Fourthly, except for mRNA level, the protein expression level of genes could also be powerful prognostic markers of patient survival. Further investigations and researches are needed to explore the relationship between the protein expression level of four genes and GC patients’ survival.

In conclusion, we developed the SERM prognostic signature related to CSC and EMT processes for predicting OS, DFS, and drug sensitivity in GC patients. Enrichment analysis to some extent unmasked a part of molecular mechanisms of the gene signature in GC, which might give guidance for developing targeted therapies. The nomogram-combined clinical characteristics and gene signature for OS prediction could improve the prognostic accuracy of the traditional TNM staging system. We anticipate that the SERM signature will offer a brand-new reference for current prognostic prediction and give more guidance in developing tailored therapy in GC patients.

## Data Availability Statement

The datasets presented in this study can be found in online repositories. The names of the repository/repositories and accession number(s) can be found in the article/**Supplementary Material**.

## Ethics Statement

Ethical review and approval was not required for the study on human participants in accordance with the local legislation and institutional requirements. Written informed consent for participation was not required for this study in accordance with the national legislation and the institutional requirements.

## Author Contributions

XJ, BC, and ZL contributed to the conception and design of the project. XJ, BC, ZL, SC, and RZ participated in the data collection, data analyses, and manuscript writing. XJ and BC made all the figures and tables. SH provided advice for the study and reviewed the manuscript. XZ and HZ edited the manuscript and supervised the project. All the authors read and approved the final manuscript. All authors contributed to the article and approved the submitted version.

## Funding

This work was funded by The National Key Research and Development Program of China (2017YFC1308900) and the Science and Technology Commission of Shanghai Municipality (grant number: STCSM18411953000) granted to HZ.

## Conflict of Interest

The authors declare that the research was conducted in the absence of any commercial or financial relationships that could be construed as a potential conflict of interest.

## Publisher’s Note

All claims expressed in this article are solely those of the authors and do not necessarily represent those of their affiliated organizations, or those of the publisher, the editors and the reviewers. Any product that may be evaluated in this article, or claim that may be made by its manufacturer, is not guaranteed or endorsed by the publisher.

## Supplementary Material

Supplementary Table S1 Clinical characteristics and mRNAsi of 296 samples in the TCGA cohort.

Supplementary Table S2 Clinical characteristics and RT-qPCR results of 126 patients in our cohort.

Supplementary Table S3 Primers of SERPINE1, EDIL3, RGS4, MATN3 and β-catenin used for RT-qPCR.

Supplementary Table S4 Univariate Cox regression analysis of 60 overlapped genes.

Supplementary Table S5 Univariate and Multivariate Cox regression of TCGA cohort with different clinical parameters and Risk score.

Supplementary Table S6 Univariate and Multivariate Cox regression of GSE66229 cohort with different clinical parameters and Risk score.

Supplementary Table S7 Univariate and Multivariate Cox regression of GSE15459 cohort with different clinical parameters and risk score.

Supplementary Figure S1 Pearson’s correlation analyses between mRNA expression level of EMT markers and risk score value in TCGA-STAD and GSE66229 cohorts.

Supplementary Figure S2 Assessment of prognostic value of gene signature on GC DFS in GSE66229 cohort. **(A)** Kaplan-Meier analysis of DFS between high- and low-risk groups based on CSCs and EMT-related gene signature. **(B)** Risk score time dependent 1-, 3-, 5- year ROC of DFS. **(C)** Multivariate Cox regression analysis of clinical parameters and risk score. **(D)** The distributions of risk score, survival status, and heatmap of CSCs and EMT-related gene signature expression levels.

Supplementary Figure S3 RT-qPCR result of SERPINE1 **(A)**, EDIL3 **(B)**, RGS4 **(C)**, MATN3 **(D)** mRNA expression levels in eight GC cell lines (HGC-27, MKN-28, SGC-7901, BGC-823, MGC-803, AGS, NCI-N87, MKN-45) and gastric mucosal cell line GES1.
