# Supplementary material for: Identification of a Four-Gene-Based SERM Signature for Prognostic and Drug Sensitivity Prediction in Gastric Cancer
Source: Front Oncol. 2022 Jan 12;11:799223. doi: 10.3389/fonc.2021.799223 (PMC8790320; doi:10.3389/fonc.2021.799223)
Supplement: Supplementary Table S1 — Clinical characteristics and mRNAsi of 296 samples in the TCGA cohort. [file DataSheet_1.zip › Table_5.docx]

**Supplementary Table 5.** Univariate and Multivariate Cox regression of TCGA-STAD cohort with different clinical parameters and Risk score.

| **Characteristics** | **Number** | **Univariate Cox**  **regression** | | **Multivariate Cox**  **regression** | |
| --- | --- | --- | --- | --- | --- |
|  |  | **Hazard**  **Ratio**  **(95%CI)** | **p-value** | **Hazard**  **Ratio** | **p-value** |
| **Age** |  |  |  |  |  |
| (＞65/≤65) | 162/134 | 1.643  (1.138-2.372) | 0.008 | 2.310  (1.563-3.416) | <0.001 |
| **Gender** |  |  |  |  |  |
| (Male/Female) | 192/104 | 1.604  (1.071-2.401) | 0.022 | 1.476  (0.977-2.229) | 0.064 |
| **Tumor stage** |  |  |  |  |  |
| II/I | 98/39 | 1.636  (0.775-3.454) | 0.197 | 1.457  (0.684-3.103) | 0.329 |
| III/I | 127/39 | 2.373  (1.176-4.792) | 0.016 | 1.903  (0.930-3.892) | 0.078 |
| IV/I | 32/39 | 4.124  (1.875-9.069) | <0.001 | 4.779  (2.135-10.699) | <0.001 |
| **T** |  |  |  |  |  |
| T2/T1 | 62/15 | 6.359  (0.858-47.127) | 0.070 |  |  |
| T3/T1 | 145/15 | 8.333  (1.156-60.094) | 0.035 |  |  |
| T4/T1 | 74/15 | 7.870  (1.074-57.676) | 0.042 |  |  |
| **N** |  |  |  |  |  |
| N1/N0 | 83/87 | 1.539  (0.914-2.589) | 0.105 |  |  |
| N2/N0 | 66/87 | 1.496  (0.859-2.608) | 0.155 |  |  |
| N3/N0 | 60/87 | 2.693  (1.609-4.508) | <0.001 |  |  |
| **M** |  |  |  |  |  |
| M1/M0 | 276/20 | 1.853  (0.958-3.515) | 0.067 |  |  |
| **Grade** |  |  |  |  |  |
| G2/G1 | 101/7 | 2.364  (0.325-17.219) | 0.396 | 2.764  (0.374-20.446) | 0.320 |
| G3/G1 | 188/7 | 3.156  (0.439-22.692) | 0.254 | 4.018  (0.548-29.440) | 0.171 |
| **Riskscore** | 296 | 2.718  (1.812-4.077) | <0.001 | 2.913  (1.920-4.419) | <0.001 |
