# Supplementary material for: Identification of a Four-Gene-Based SERM Signature for Prognostic and Drug Sensitivity Prediction in Gastric Cancer
Source: Front Oncol. 2022 Jan 12;11:799223. doi: 10.3389/fonc.2021.799223 (PMC8790320; doi:10.3389/fonc.2021.799223)
Supplement: Supplementary Table S1 — Clinical characteristics and mRNAsi of 296 samples in the TCGA cohort. [file DataSheet_1.zip › Table_6.docx]

**Supplementary Table 6.** Univariate and Multivariate Cox regression of GSE66229 cohort with different clinical parameters and Risk score.

| **Characteristics** | **Number** | **Univariate Cox**  **regression** | | **Multivariate Cox**  **regression** | |
| --- | --- | --- | --- | --- | --- |
|  |  | **Hazard**  **Ratio**  **(95%CI)** | **p-value** | **Hazard**  **Ratio** | **p-value** |
| **Age** |  |  |  |  |  |
| (＞65/≤65) | 172/128 | (1.138-2.372) | 0.008 | 1.871  (1.347-2.599) | <0.001 |
| **Gender** |  |  |  |  |  |
| (Male/Female) | 199/101 | 1.604  (1.071-2.401) | 0.022 | 1.133  (0.801-1.603) | 0.480 |
| **Tumor stage** |  |  |  |  |  |
| II/I | 97/30 | 1.636  (0.775-3.454) | 0.197 | 1.621  (0.625-4.202) | 0.320 |
| III/I | 96/30 | 2.373  (1.176-4.792) | 0.016 | 3.240  (1.273-8.244) | 0.014 |
| IV/I | 77/30 | 4.124  (1.875-9.069) | <0.001 | 8.745  (3.454-22.140) | <0.001 |
| **T** |  |  |  |  |  |
| T3/T2 | 91/186 | 2.337  (0.858-47.127) | <0.001 |  |  |
| T4/T2 | 21/186 | 2.480  (1.443-4.261) | <0.001 |  |  |
| **N** |  |  |  |  |  |
| N1/N0 | 131/38 | 1.743  (0.858-3.539) | 0.124 |  |  |
| N2/N0 | 80/38 | 3.367  (1.654-6.854) | <0.001 |  |  |
| N3/N0 | 51/38 | 6.942  (3.363-14.329) | <0.001 |  |  |
| **M** |  |  |  |  |  |
| M1/M0 | 273/27 | 3.84  (2.482-5.942) | 0.067 |  |  |
| **Lauren** |  |  |  |  |  |
| Mixed/Intestinal | 19/146 | (0.325-17.219) | 0.396 | 2.625  (1.456-4.832) | 0.002 |
| Diffuse/Intestinal | 135/146 | 3.156  (0.439-22.692) | 0.254 | 1.388  (0.978-1.973) | 0.068 |
| **Riskscore** | 300 | 121.011  (21.175 - 691.553) | <0.001 | 123.2903  (18.181-836.087) | <0.001 |
