# Supplementary material for: Identification of a Four-Gene-Based SERM Signature for Prognostic and Drug Sensitivity Prediction in Gastric Cancer
Source: Front Oncol. 2022 Jan 12;11:799223. doi: 10.3389/fonc.2021.799223 (PMC8790320; doi:10.3389/fonc.2021.799223)
Supplement: Supplementary Table S1 — Clinical characteristics and mRNAsi of 296 samples in the TCGA cohort. [file DataSheet_1.zip › Table_7.docx]

**Supplementary Table 7.** Univariate and Multivariate Cox regression of GSE15459 cohort with different clinical parameters and Risk score.

| **Characteristics** | **Number** | **Univariate Cox**  **regression** | | **Multivariate Cox**  **regression** | |
| --- | --- | --- | --- | --- | --- |
|  |  | **Hazard**  **Ratio**  **(95%CI)** | **p-value** | **Hazard**  **Ratio** | **p-value** |
| **Age** |  |  |  |  |  |
| (＞60/≤60) | 133/59 | 0.983  (0.641-1.506) | 0.936 | 1.606  (0.997-2.588) | 0.052 |
| **Gender** |  |  |  |  |  |
| (Male/Female) | 125/67 | 1.402  (0.908-2.165) | 0.127 | 0.780  (0.490- 1.243) | 0.296 |
| **Tumor stage** |  |  |  |  |  |
| II/I | 29/31 | 2.396  (0.738-7.782) | 0.146 | 1.934  (0.587-6.368) | 0.278 |
| III/I | 72/31 | 7.855  (2.797-22.057) | <0.001 | 6.828  (2.384-19.555) | <0.001 |
| IV/I | 60/31 | 20.498  (7.202-58.338) | <0.001 | 19.715  (6.643-58.507) | <0.001 |
| **Lauren** |  |  |  |  |  |
| Mixed/Intestinal | 18/99 | 0.873  (0.574-1.328) | 0.526 | 0.468  (0.209-1.047) | 0.065 |
| Diffuse/Intestinal | 75/99 | 0.672  (0.301-1.499) | 0.331 | 0.948  (0.597-1.505) | 0.820 |
| **Riskscore** | 300 | 3.766  (2.257-6.283) | <0.001 | 2.432  (1.451-4.076) | <0.001 |
