# Supplementary figures and images for: Identification of a Four-Gene-Based SERM Signature for Prognostic and Drug Sensitivity Prediction in Gastric Cancer
Source: Front Oncol. 2022 Jan 12;11:799223. doi: 10.3389/fonc.2021.799223 (PMC8790320; doi:10.3389/fonc.2021.799223)

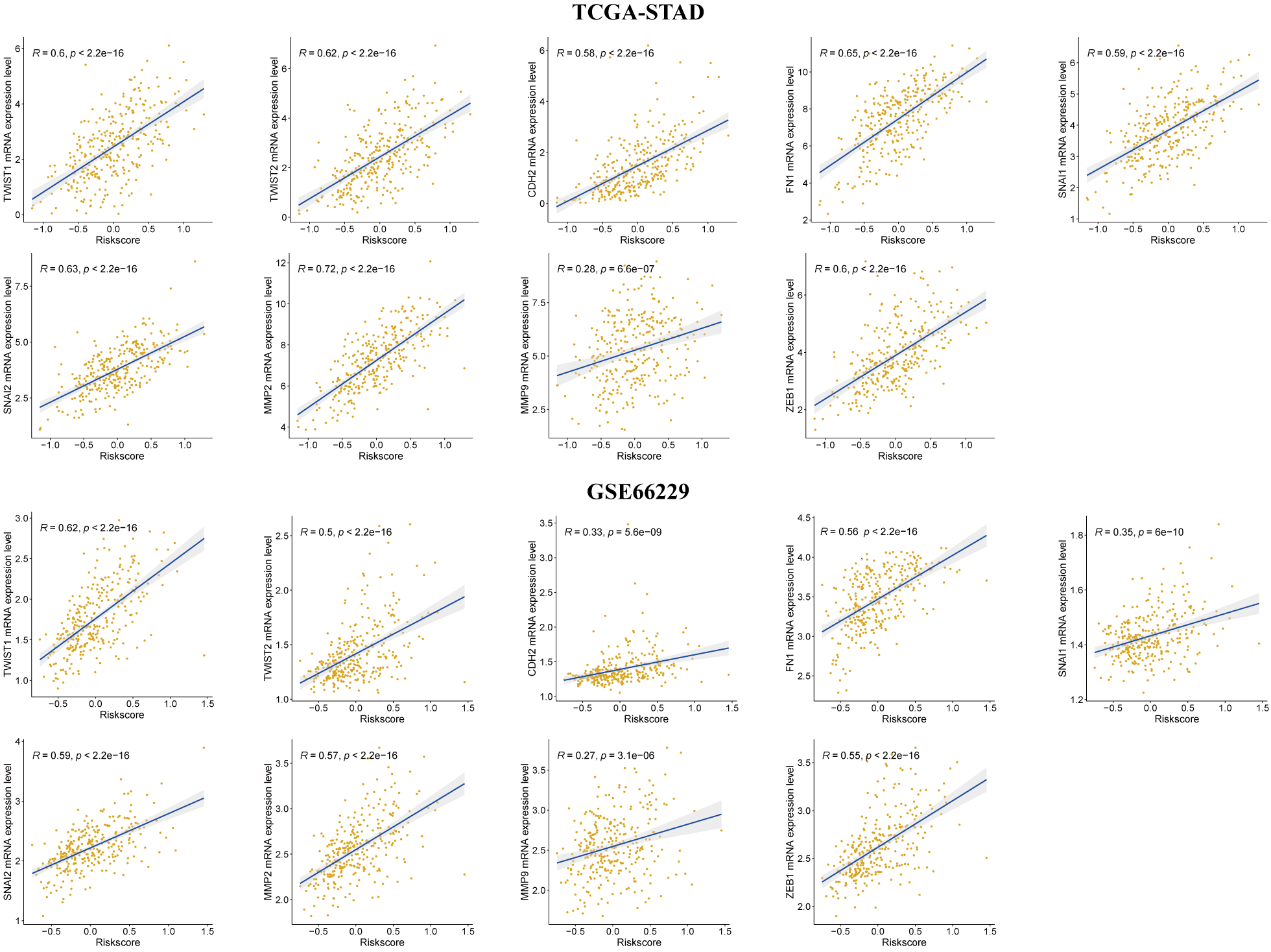

Supplement: Supplementary Table S1 — Clinical characteristics and mRNAsi of 296 samples in the TCGA cohort. [file DataSheet_1.zip › Image_1.tif]

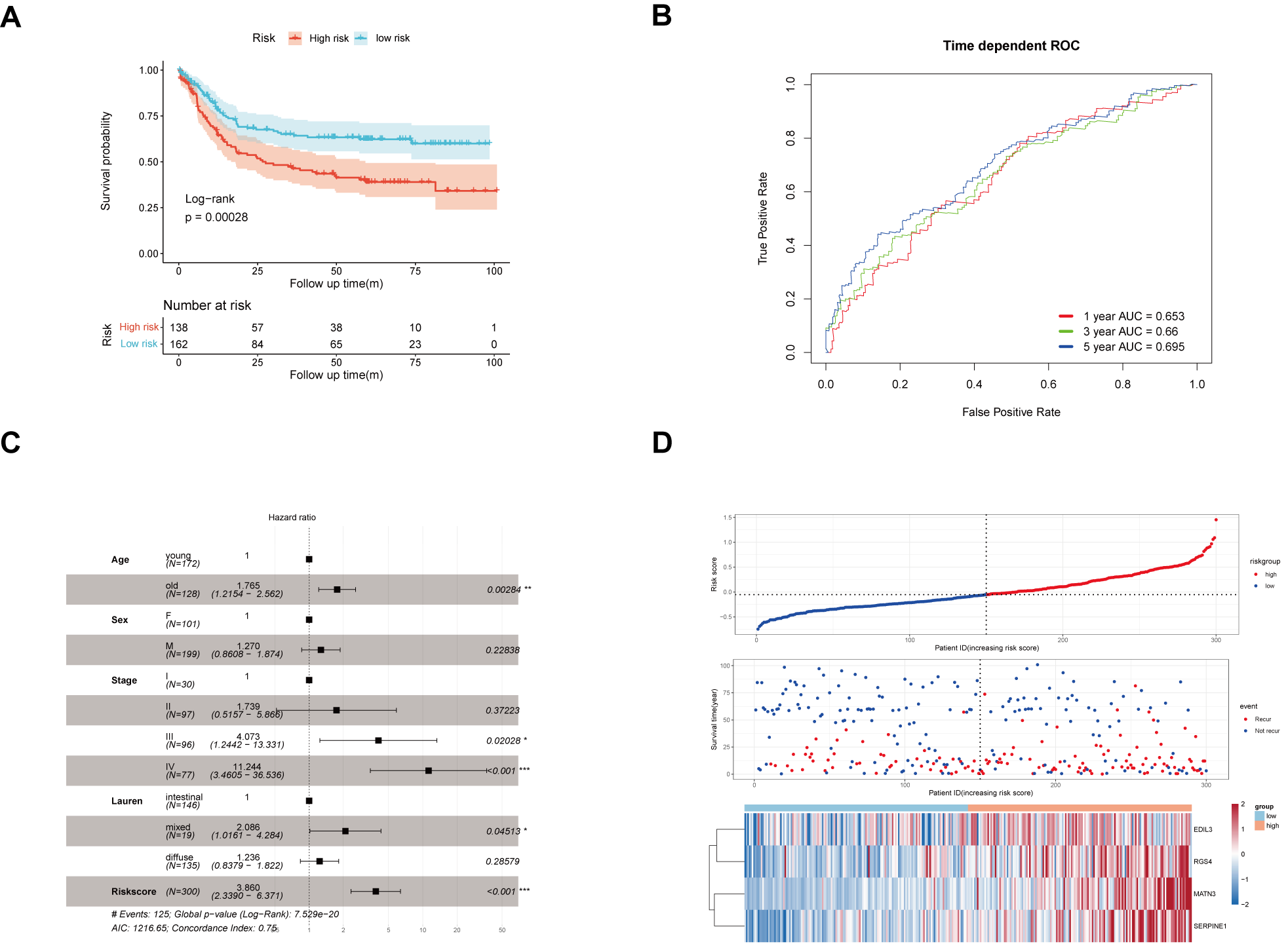

Supplement: Supplementary Table S1 — Clinical characteristics and mRNAsi of 296 samples in the TCGA cohort. [file DataSheet_1.zip › Image_2.tif]

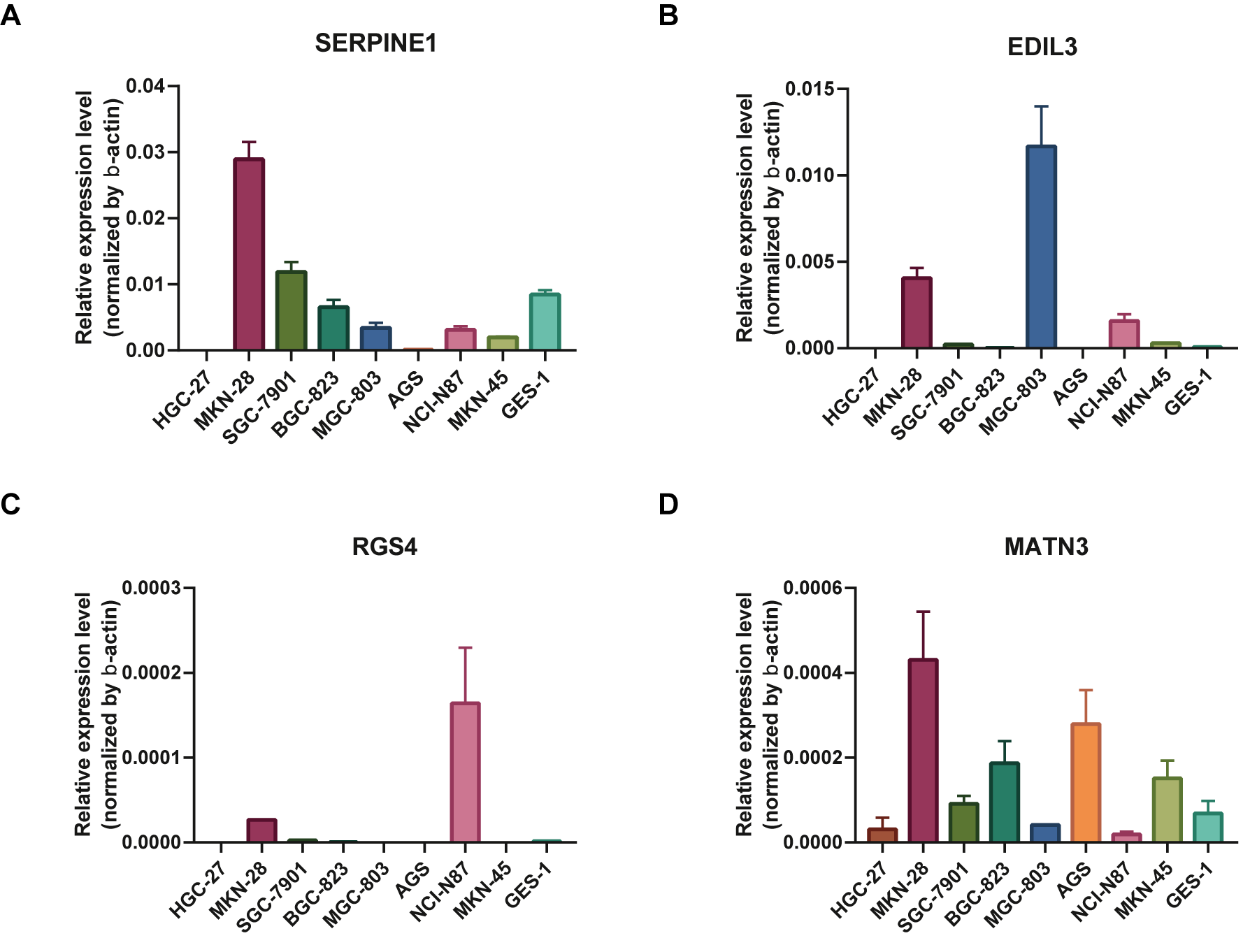

Supplement: Supplementary Table S1 — Clinical characteristics and mRNAsi of 296 samples in the TCGA cohort. [file DataSheet_1.zip › Image_3.tif]
